# Evaluating the Use of Online Self-Report Questionnaires as Clinically Valid Mental Health Monitoring Tools in the Clinical Whitespace

**DOI:** 10.1007/s11126-023-10022-1

**Published:** 2023-05-05

**Authors:** Kaitlyn Arrow, Philip Resnik, Hanna Michel, Christopher Kitchen, Chen Mo, Shuo Chen, Carol Espy-Wilson, Glen Coppersmith, Colin Frazier, Deanna L. Kelly

**Affiliations:** 1 Maryland Psychiatric Research Center, University of Maryland School of Medicine, Catonsville, MD USA; 2 Institute for Advanced Computer Studies, Department of Linguistics, University of Maryland College Park, College Park, MD USA; 3 Johns Hopkins University, Baltimore, MD USA; 4 Institute for Systems Research, University of Maryland College Park, College Park, MD USA; 5 Maryland Psychiatric Research Center, University of Maryland School of Medicine, PO Box 21247, Baltimore, MD 21228 USA

**Keywords:** Depression, Schizophrenia, Internet, Self-report, Technology

## Abstract

Although digital health solutions are increasingly popular in clinical psychiatry, one application that has not been fully explored is the utilization of survey technology to monitor patients outside of the clinic. Supplementing routine care with digital information collected in the “clinical whitespace” between visits could improve care for patients with severe mental illness. This study evaluated the feasibility and validity of using online self-report questionnaires to supplement in-person clinical evaluations in persons with and without psychiatric diagnoses. We performed a rigorous in-person clinical diagnostic and assessment battery in 54 participants with schizophrenia (N = 23), depressive disorder (N = 14), and healthy controls (N = 17) using standard assessments for depressive and psychotic symptomatology. Participants were then asked to complete brief online assessments of depressive (Quick Inventory of Depressive Symptomatology) and psychotic (Community Assessment of Psychic Experiences) symptoms outside of the clinic for comparison with the ground-truth in-person assessments. We found that online self-report ratings of severity were significantly correlated with the clinical assessments for depression (two assessments used: R = 0.63, p < 0.001; R = 0.73, p < 0.001) and psychosis (R = 0.62, p < 0.001). Our results demonstrate the feasibility and validity of collecting psychiatric symptom ratings through online surveys. Surveillance of this kind may be especially useful in detecting acute mental health crises between patient visits and can generally contribute to more comprehensive psychiatric treatment.

**Supplementary Information:**

The online version contains supplementary material available at 10.1007/s11126-023-10022-1.

## Introduction

In an age where the need for mental health services far outweighs the number of providers available, exploring new ways that allow clinicians to serve more patients is vital. In 2017, one in five adults experienced a mental illness [1], and in 2018, it was reported that 56% of adults with a mental illness never received any treatment [2]. While many of these people may have never sought out help, one in five adults with a mental illness that did seek help never received requisite care [2]. Part of this gap in treatment comes from a lack of access to care. According to the National Institute on Mental Illness (NAMI), 55% of counties in the U.S. do not have a practicing psychiatrist [1]. In counties that do, 2020 data indicate that many psychiatrists contacted were either not accepting new patients or did not accept their insurance plan [3]. For those able to schedule an appointment, average wait times range from three to ten weeks, and some clinics have wait times of over one year [4, 5]. Additionally, 68% of psychiatrists reported that their waitlists have grown even longer since the beginning of the coronavirus (COVID-19) pandemic [6]. Waiting for an appointment with a psychiatrist or mental health professional can be detrimental for those living with a serious mental illness or at a high risk for suicidal behaviors.

For those able to consistently see a psychiatrist, 15 to 20 min appointments are typical, and a period of one to three months is the average time between visits [7]. This interval between healthcare interactions, or the “clinical whitespace” [8], prevents clinicians from gathering up-to-date information on their patients.

To understand the detrimental effects that lack of health care access and stigma can have on an individual, Stene-Larsen and Reneflot [9] reviewed literature that reported the rates of contact individuals that died by suicide had with health care professionals from 2000 to 2017. The authors found that among those who died by suicide, 80% had contact with primary health care services in the year leading up to their death, and 57% had contact within one month of their suicide. The rates of contact individuals had with mental health professionals specifically within a year of their death was only 31%, and only 21% had contact with a mental health professional within a month of their suicide.

Evidently, many people who are in great need of mental health care lack access to that care and are left unmonitored during times of crises. Even for individuals who can access primary or mental health care services, traumatic events and symptom exacerbation occurs in this clinical whitespace and may go unnoticed by health care professionals. Stene-Larsen and Reneflot [9] show that this lack of access to mental health care professionals in particular - whether it be between appointments or having no access at all - can have fatal effects. Currently, clinicians have few ways to assess the status and symptoms of individuals who are not physically in front of them. With the pressing need to clinically evaluate more people than is possible for mental health professionals to see in-person, the ability to monitor and assess individuals outside of the office is vital.

One way to meet this growing need is by using technology. Harnessing online data, capitalizing on better ways to share information, and real time assessments using smart technology are becoming a fixture of our society. Some clinicians use technological tools, such as online self-report questionnaires, to gather more information on the client before an appointment. These questionnaires allow people to quickly, and without aid from a clinician, report their symptoms for diagnosis or evaluation.

Self-report questionnaires are powerful clinical tools because they are often validated and allow the patient to give their first perspective. Even so, they still have potential problems, such as social desirability and recall biases [10], lack of trust, or lack of insight leading to incomplete or inaccurate reporting [11]. Online self-report may represent a unique tool for assessing patient data in real time and at more convenient times for patients, however, less is known about how well online data for assessing psychiatric symptoms, particularly in illnesses where prioritization for immediate care may be needed such as depression or schizophrenia, may correspond to clinician rated symptoms. Some have wondered if patients may respond differently online due to worries over security of their data [12] and their comfortability with using technology [13]. Others have wondered if personal expression online and in-person would correspond [14].

To try to address these questions, some studies have tested for differences in the psychometric properties of online assessments and their pencil and paper predecessors. For many questionnaires, such studies have found strong correlations between the online and in-person formats. For example, many researchers feel confident that the online version of the Montgomery-Asberg Depression Rating Scale [15] is as effective as it is on paper [16–18].

Since in-person self-report questionnaires are validated against in-person clinician evaluations, and online questionnaires validated against in-person questionnaires, it is a seemingly safe assumption that, through this process, the validity of online self-report questionnaires would be significantly correlated with in-person clinical assessments for depression and psychosis. However, we have been unable to identify any studies that test this assumption.

With online and self-reported questionnaires becoming increasingly integrated in health care, there still is a gap in the literature explicating how technology can be used to collect clinically valid data in the clinical whitespace. In this paper, we begin to fill in that gap by studying the relationship of online self-report questionnaire data collected outside of the clinic using an online platform, compared to a ground truth in-person evaluation in a clinic. We chose to focus on depressive and psychotic symptoms for proof-of-concept purposes, as such symptoms are highly prevalent and are associated with greater risk for mortality and suicidal behaviors. Depression and schizophrenia thus represent high public health priority diseases, and are considered two of the greatest contributors to the global disability-adjusted life year burden [19].

## Methods

### Participants and Recruitment

This work is part of a larger project where we have collected online data (including online questionnaire responses, social media, or both) from willing and consenting participants using a web-based data donation platform, umd.ourdatahelps.org. The focus of our project is to explore the utilization of machine learning techniques to identify signals in language to help prioritize mental health care in people with worsening symptoms of schizophrenia and depression [20]. We have heavily recruited for individuals with mental health conditions, resulting in a dataset of 4,134 participants where 63% of participants have a self-reported psychiatric diagnosis. All these participants were willing to consent and give their donated mental health data to date. This study concerns a subset of 54 participants that were actively recruited and enrolled in the larger online study (under the domain umd.ourdatahelps.org), but also concomitantly completed a series of in-clinic assessments. Those 54 participants comprise three diagnostic groups (schizophrenia, depression, and health controls), and the in-person and online assessments were completed on the same day. These in-person assessments were comprehensive clinical interviews by highly trained personnel at the Maryland Psychiatric Research Center (MPRC), University of Maryland School of Medicine. The findings reported from this project can help us and others determine if it is feasible and valid to use online reported depressive and psychotic symptoms.

Participants were recruited through clinics, inpatient programs, and recontact of those with previous research participation. Additionally, we recruited through local newspaper advertisements and flyers at local universities. The study was approved by the University of Maryland Institutional Review Board and all participants provided informed consent. These 54 participants were recruited, and data was collected, between March of 2017 and December of 2018.

### Procedures

Trained and reliable clinical raters conducted a semi-structured in-person clinical interview for each of the 54 participants to assess depressive and psychotic symptoms. The raters are highly trained clinical professionals who regularly participate in reliability and practice sessions and maintain an inter-rater correlation coefficient (ICC) greater than 0.8 with a consensus gold standard rating created by a team of experts; this meets or exceeds the highest standards for clinical ratings worldwide.

The Structured Clinical Interview for Diagnosis (SCID) [21] was used to validate and confirm the individual’s diagnosis. In the semi-structured interview, we used the Montgomery-Asberg Rating Scale (MADRS) [15], a 10-question clinical evaluation scale rating depressive symptoms from zero to six, the Hamilton Depression Rating Scale (HDRS) [22], a 17-question scale to rate depressive symptoms from zero to four; and the Brief Psychiatric Rating Scale (BPRS) [23], an 18-question clinical evaluation scale to rate general, positive, and negative symptoms of schizophrenia from one to seven. For the BPRS, we also used the Psychosis Subfactor section, which contains 4 items involving suspiciousness, unusual thought content, conceptual disorganization, and hallucinations for the evaluation of positive symptoms. While in the clinic, we also had participants complete the Quick Inventory of Depressive Symptoms (QIDS-SR) [24], a 16-question self-report measure rating depressive symptoms from zero to three.

During the clinic visit, participants were enrolled in the umd.ourdatahelps.org portal and were asked to complete self-report questionnaires online at home later that same day. The QIDS-SR for depressive symptoms and the CAPE-15 for psychotic symptoms, both short and validated instruments, were administered online to determine if the symptoms corresponded with the gold standard rating scales above.

### Statistical Analysis

The analyses were carried out using R (version 4.1.1). Pearson correlation was calculated to assess the association between the online and in-person measurements. The correlation analyses were performed between two online measurements (QIDS-SR and CAPE-15) and in-person measurements including QIDS-SR, HDRS, MADRS, and BPRS. False discovery rate (FDR) correction was used to account for the multiple testing.

## Results

### Demographic Information

Table 1 lists the demographic information of the 54 participants including gender, age, and race. The mean age of participants was 36 years old with a similar breakdown by gender and race. The larger dataset of over 4000 participants has a very similar gender and age characterization (data not shown).

**Table 1:** Participant Characteristics by Diagnosis Group

Characteristic	All N = 54	Schizophrenia N = 23	Depression N = 14	Healthy Controls N = 17
Age Mean (SD), years	36.31 ± 11.42	38.48 ± 11.67	36.43 ± 14.28	22.29 ± 11.96
**Gender** Male Female	29 (54%) 25 (46%)	16 (70%) 7 (30%)	2 (14%) 12 (86%)	11 (65%) 6 (35%)
**Ethnicity** Hispanic Non-Hispanic	2 (4%) 52 (96%)	1 (4%) 22 (96%)	0 14 (100%)	1 (6%) 16 (94%)
**Race** African American Caucasian Asian More than one Race	24 (44%) 24 (44%) 5 (9%) 1 (2%)	15 (65%) 8 (35%) 0 0	5 (36% 7 (50%) 1 (14%) 0	4 (24%) 9 (53%) 3 (18%) 1 (6%)

### Validation of Online and In-Person Self-Reports

**Fig. 1:**
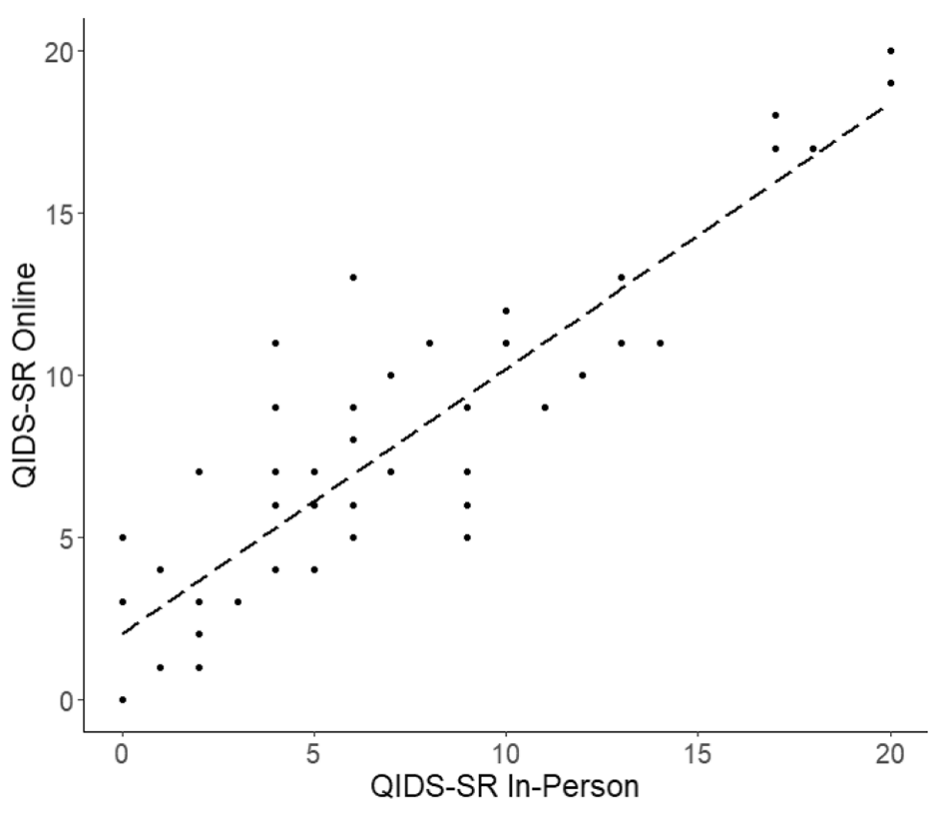
Plot of Online and In-Person QIDS-SR

First, we tested reliability and rating consistency by comparing the self-report data from our online questionnaires with the self-reports completed in person. For the QIDS-SR, the correlation between the same-day online and in-person scores for the sample was R = 0.88, p < 0.0001, and FDR-adjusted p-value (p*) < 0.0001 (Fig. 1).

### Validation for Depressive Symptoms

**Fig. 2:**
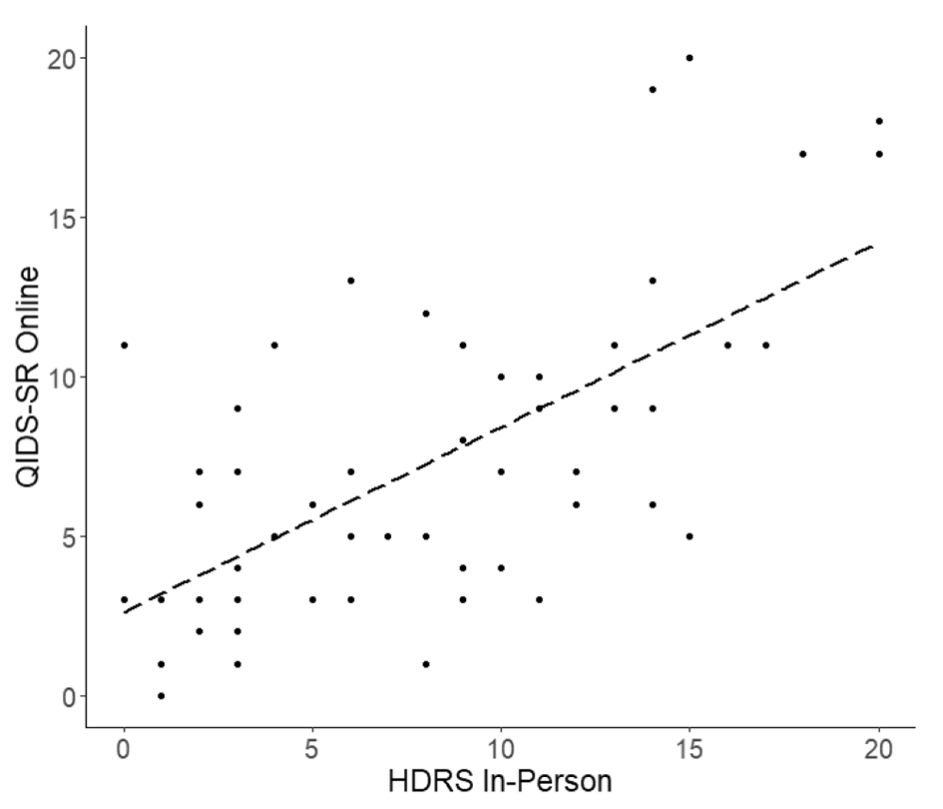
Plot of Online QIDS-SR with HDRS

**Fig. 3:**
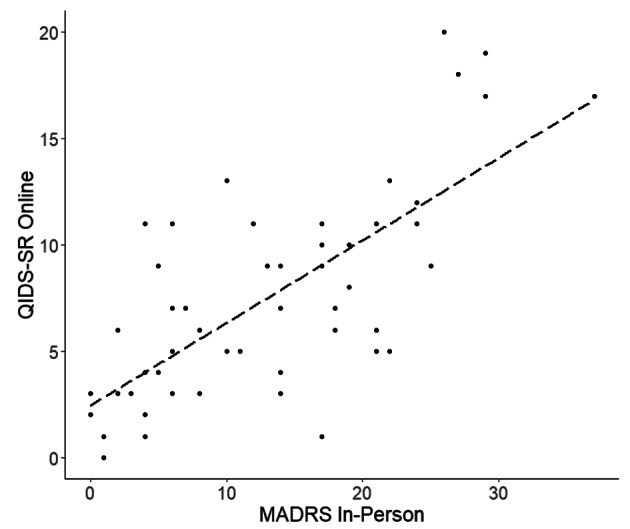
Plot of Online QIDS-SR with MADRS.

econd, we compared the same online QIDS-SR responses with clinical ratings for depressive symptoms as determined by our trained rater based on in-person clinical interviews using two depression rating scales. Figure 2 illustrates the correlations of how all participants rated themselves on the QIDS-SR and how the trained rater scored them with the HDRS (R = 0.66, p < 0.0001, p* <0.0001) and Fig. 3 shows the QIDS-SR correlation with the MADRS (R = 0.73, p < 0.0001, p* <0.0001).

### Validation for Psychotic Symptoms

**Fig. 4:**
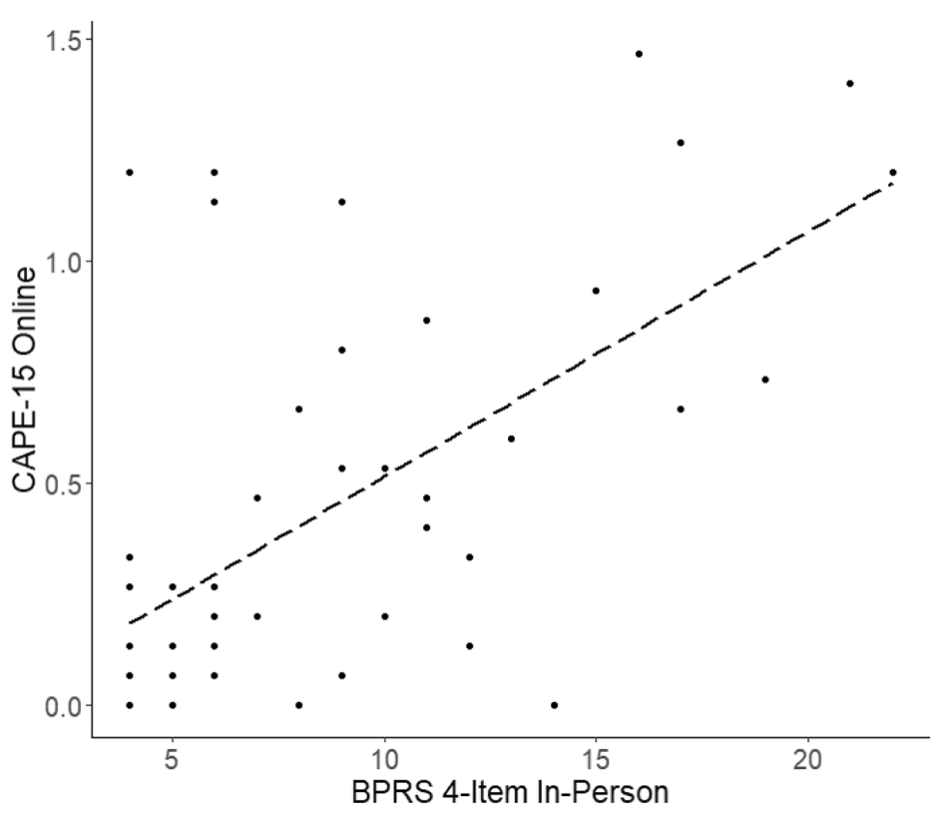
Plot of Self-Report CAPE-15 with In-Person Psychosis Subfactor of BPRS

Results for psychotic symptoms are similar. As illustrated in Fig. 4 we found a significant correlation between the CAPE-15 self-report scores for psychotic symptoms and the clinical evaluation from our rater on the four-item psychosis subfactor section of the gold standard BPRS among all participants (R = 0.62, p < 0.0001, p* <0.0001). This relationship is only significant in the schizophrenia group (data not shown, R = 0.63, p = 0.0012, p* =0.0030).

## Discussion

This work expands upon current literature by showing that self-report data on depressive and psychotic symptoms donated to an online data portal system are found to be similar to clinician symptom assessments performed by raters in a clinical setting. Evidence such as this, that supports the integration of technological platforms to help validate online self-report questionnaires, has been currently lacking and shows that symptom severity can be reliably determined despite using different rating instruments. In fact, the purpose of the study was not to validate specific rating instruments for direct clinical use, but to evaluate if online modes of data collection could provide meaningful signals of valid snapshots of depressive and psychotic symptomatology. Thus, our research indicates that online self-report assessments may represent a different, but equally valid, environment for patients to share their experiences outside a clinical setting.

While the findings from our study are very promising, there are limitations to our work. First, participants in our study were compensated with a $15 gift card to fill out online questions when prompted. However, we believe that people are willing to donate online data with little to no incentives – data which could be stored and used for future analysis. Second, we had a relatively small sample size of N = 54, however such participants underwent an extensive inclusion process, had confirmed clinical diagnoses, and comprehensive structured interviews – thus, the quality of the data was high. Others have validated self-report data against clinical interviews, but such investigations are not structured as online vs. in person, and they do not comprise participants of varying diagnostic groups [25–27]. Another potential limitation is that our choice of online psychometric measures (QIDS-SR, CAPE-15) are not commonly used in primary care settings. However, we designed this to be a study strength, as the main purpose of this investigation was to ascertain a valid signal of depressive and psychotic symptoms when monitoring participants remotely, not to replace or stand-in as primary care assessments to help screen for symptoms. All measures used in this study, both in-person and online, are validated instruments being used for proof-of-concept purposes only and are not intended to comprise an ideal assessment battery for clinical use.

Further limitations of our study include the possibility of volunteer subject bias, in which the characteristics of those who volunteered meaningfully differ from those who chose not to participate. Additionally, the individuals who participated in this pilot study were recruited differently from the thousands of participants who only donated social media data and completed the self-report questionnaires. This could magnify the potential volunteer bias which could reduce the generalizability of these results. However, we did examine the sample demographic information of the 4,134 enrolled in the online portal and found a similar age, race, and sex distribution to the sample we present.

Although we were able to obtain accurate and valid ratings for people with schizophrenia, depression, and those without a psychiatric diagnosis, further research is needed to verify that these findings can expand to other psychiatric diagnoses such as anxiety disorders, autism spectrum disorders, or personality disorders.

## Conclusions

While self-report measures should never replace in-person clinical evaluations, they can provide valuable supplemental information to clinicians and will likely be used in the future. Clinicians could use online self-report measures in the clinical whitespace, allowing them to receive clinically valid information during a period where they are not able to see the patient. Clinicians can better manage patient volume by administering online self-report measures to those who are seeking an intake appointment, thereby quickly determining severity. This would allow clinicians to prioritize those dealing with more severe symptoms and possibly provide life-saving intervention to clients waiting to see a clinician in-person.

While many people use simple screening tools in depression such as the Patient Health Questionnaire-9 (PHQ-9), such assessments are screening instruments rather than tools to measure change over time. We selected a brief instrument for depressive symptoms for this study that could show change. In fact, Trivedi and colleagues [24] recommended that clinicians use the QIDS-SR as a measurement, “for use in daily practice to provide a guide for clinicians by which to better gauge the effects of treatment and therefore to enable a timely revision in the treatment plan when needed” (p. 74). Whether it is the QIDS-SR or other instruments, moving to providing self-reports online should be a natural step for clinicians, particularly when in person meetings may be less available. New platforms such as MindLogger are emerging for this purpose [28]. These technologies could alert and provide clinicians with valid mental health signals in the clinical whitespace that were previously inaccessible.

While more work is needed to confirm the strength and generalizability of our conclusions, the present results help show the validity of data from online self-reports to assist clinicians monitor their patients. The ability to monitor patients outside of the clinic will allow clinicians to see more individuals in need, feel more confident in online information gathering, and notice an individual’s exacerbation of symptoms to provide life-saving interventions that may not have been possible before.

## Electronic Supplementary Material

Below is the link to the electronic supplementary material.

Supplementary Material 1
