# Supplementary material for: Evaluating the Use of Online Self-Report Questionnaires as Clinically Valid Mental Health Monitoring Tools in the Clinical Whitespace
Source: Psychiatr Q. 2023 May 5;94(2):221–31. doi: 10.1007/s11126-023-10022-1 (PMC10160731; doi:10.1007/s11126-023-10022-1)
Supplement: Supplementary file 1 — Supplementary Material 1 [file 11126_2023_10022_MOESM1_ESM.docx]

Conflicts of Interest

Dr. Deanna Kelly has served as a consultant for Alkermes, Janssen, and Sunovion, with this role unrelated to the present work. The other authors have nothing to disclose with the exception of Dr. Glen Coppersmith who worked for Qnfty at the time of the study and now works for SonderMind.
